# The development of a performance evaluation index system for Chinese Centers for Disease Control and Prevention: a Delphi consensus study

**DOI:** 10.1186/s41256-024-00367-w

**Published:** 2024-07-23

**Authors:** Huimin Sun, Ying Wang, Huanle Cai, Pengyu Wang, Jie Jiang, Congxing Shi, Yongyue Wei, Yuantao Hao

**Affiliations:** 1 Department of Epidemiology and Biostatistics, School of Public Health, Peking University, Beijing, China; 2 Department of Medical Statistics, School of Public Health, Sun Yat-Sen University, Guangzhou, China; 3 Peking University Center for Public Health and Epidemic Preparedness & Response, Beijing, China; 4 Key Laboratory of Epidemiology of Major Diseases (Peking University), Ministry of Education, Beijing, China

**Keywords:** Delphi method, Performance evaluation, CDC, Public health, Index system

## Abstract

**Background:**

The performance evaluation of the Centers for Disease Control and Prevention (CDC) is crucial for enhancing the quality of public health services. With the ongoing reform of the CDC system in China, the existing performance evaluation system faces challenges. This study used the Delphi method to develop a new performance evaluation system for China’s provincial, city, and county-level CDC.

**Methods:**

Following the “Structure-Process-Outcome” model, assessment indicators were systematically collected. Indicators were modified and screened through two Delphi rounds based on CDC responsibilities, health development, and national policies. Twenty-four experts provided ratings and recommendations, and the research team evaluated questionnaire reliability, expert positivity, expert authority, and opinion consistency.

**Results:**

The preliminary index system identified through the literature review and pre-survey included 11 primary, 30 secondary, and 64 tertiary indicators. After the first round of consultation, two secondary indicators and 11 tertiary indicators were removed and 22 tertiary indicators were added. After the second round of consultation, three secondary indicators and 11 tertiary indicators were removed and three tertiary indicators were added, at which point the *p*-value of the test for Kendall’s coefficient of concordance W was < 0.001 and the coefficient of variation was within acceptable limits (< 0.25), so the consultation was concluded. The final index system included 11 primary, 25 secondary, and 67 tertiary indicators.

**Conclusions:**

This study responded to the CDC system reform by developing a comprehensive performance evaluation index system for provincial, city, and county-level CDC in China. The index system is both scientifically grounded and practical, serving as an effective tool for promoting the high-quality work of CDC organizations.

**Supplementary Information:**

The online version contains supplementary material available at 10.1186/s41256-024-00367-w.

## Introduction

The COVID-19 epidemic has highlighted Chinese Centers for Disease Control and Prevention's (CDC) critical role in crisis response while showcasing the challenges they encountered in managing major infectious disease outbreaks and public health emergencies. The work and efforts of Chinese CDC during this epidemic have provided an opportunity for future reforms. Performance evaluation of CDC is an essential means of scientifically evaluating the implementation of public health work, promoting CDC institutions at all levels to fulfill their primary responsibilities, and improving work performance and service quality [1, 2]. The Chinese CDC system comprises four levels: national, provincial, city, and county. At the apex, China CDC is tasked with furnishing authoritative scientific evidence and technical support for pivotal issues in disease prevention and control, alongside the formulation of pertinent work standards. Provincial CDC institutions are mandated to devise and execute effective strategies tailored to their respective regions, and to oversee and evaluate their subordinate CDC entities to ensure the quality and efficacy of prevention and control efforts. City CDC institutions are equipped with robust field investigation and emergency response capabilities that enable timely detection and management of public health crises. County CDC institutions collaborate closely with community health institutions, focusing on enhancing their capacity and fortifying the grassroots foundation of disease prevention. The structured and functional approach enables China’s four-tier CDC system to adeptly implement disease prevention and control measures, thereby safeguarding public health. In 2021, the National Disease Control and Prevention Administration was officially inaugurated, marking the beginning of the reform of Chinese CDC system. Correspondingly, the performance evaluation standards of CDC institutions should be adjusted and updated [3].

The performance evaluation of Chinese CDC institutions officially began in 2008, and was revised and improved in 2012 and 2015, respectively [4–6]. In 2015, the National Health and Family Planning Commission issued the “*Performance Evaluation Index System for Disease Prevention and Control Institutions (Trial)*”, which has been in continuous use since then [6]. Performance evaluation criteria should be appropriate to CDC’s functions and the current health development situation. However, as time progressed, the current performance evaluation system has encountered several issues. First, some indicators are beyond the scope of CDC’s functions [7, 8]. For example, in the case of the “Completion rate of occupational disease reporting”, quite a number of CDC institutions lack the function of diagnosing and reporting occupational diseases in practice [9]. Second, the current index system synthesizes evaluation indicators into four domains, comprising 15 secondary and 35 tertiary indicators. This scheme appears to be less comprehensive than leading international public health bodies. Notably, the evaluation systems of the World Health Organization (WHO), the United States, and the United Kingdom explicitly prioritize health financing [10–12]. This critical but rudimentary function involves the mobilization and allocation of funds for health services and ensures universal access to effective public and private health care [13, 14]. In recent years, the Chinese government has significantly increased its investment in disease control and prevention, while the 2015 index system lacks any assessment of funding [15, 16]. Similarly, the frameworks of the WHO and the United States for health systems performance assessment underscore the crucial role of the health workforce, recognizing that system performance depends on the competencies, skills, and motivation of those professionals delivering services [11, 12]. In China, however, there is still a shortage of CDC professionals, and the existing performance evaluation system does not cover the assessment of professional and technical staff [8, 17–19]. Third, the current evaluation system cannot adapt to the continuous development of health in China. The COVID-19 outbreak has highlighted the importance of laboratory testing, major infectious disease surveillance and early warning, and public health crisis emergency response, which are also priorities for China’s health development during the 14th Five-Year Plan [20]. Therefore, consideration should be given to an increase in the number of such evaluation indicators [3, 21–24]. Besides, the *Vaccine Administration Law of the People’s Republic of China* requires the implementation of a full electronic vaccine traceability system and calls for strengthening vaccine management [25], but this aspect is not reflected in the 2015 index system. These challenges have resulted in subpar performance evaluation outcomes in some areas, hindering efforts to drive system reform and modernization within CDC institutions [3].

In this study, we systematically gathered performance evaluation indicators of CDC and established a performance evaluation framework. Then, we employed the Delphi expert consultation to evaluate and screen the indicators. Finally, a scientific and reasonable performance evaluation index system was formulated. It is expected to provide guidance for the high-quality implementation of the performance evaluation for CDC institutions, and to reflect the direction for the reform and modernization of Chinese CDC systems at the provincial, city, and county levels.

## Methods

### Establishment of the performance evaluation framework and collection of indicators

This study adopted the “Structure-Process-Outcome” (SPO) theoretical model as the fundamental framework for evaluating the quality of public health services. The SPO model was proposed by Donabedian in 1966 and is now widely used in the quality evaluation of healthcare services [26, 27]. Based on the current requirements for the operational work of Chinese CDC, we divided subcategories under the three dimensions of the SPO model, which constitute the primary indicators in the index system. Subsequently, by reviewing relevant studies and policy documents on performance evaluation in Web of Science, PubMed, China National Knowledge Infrastructure, and the official CDC websites at home and abroad, we systematically collected the evaluation indicators. The criteria for indicator inclusion were as follows: (1) all indicators of the “*Performance Evaluation Index System for Disease Prevention and Control Institutions (Trial)*” [6], (2) indicators that were of concern to the national policy after the COVID-19, such as those about infectious disease prevention and control and emergency response, and (3) indicators reported in literature and policy documents related to primary CDC operations [25]. All the specific indicators collected were organized into the framework of the performance evaluation index system and further subdivided into secondary and tertiary indicators.

### Delphi method and its implementation process

**Fig. 1:**
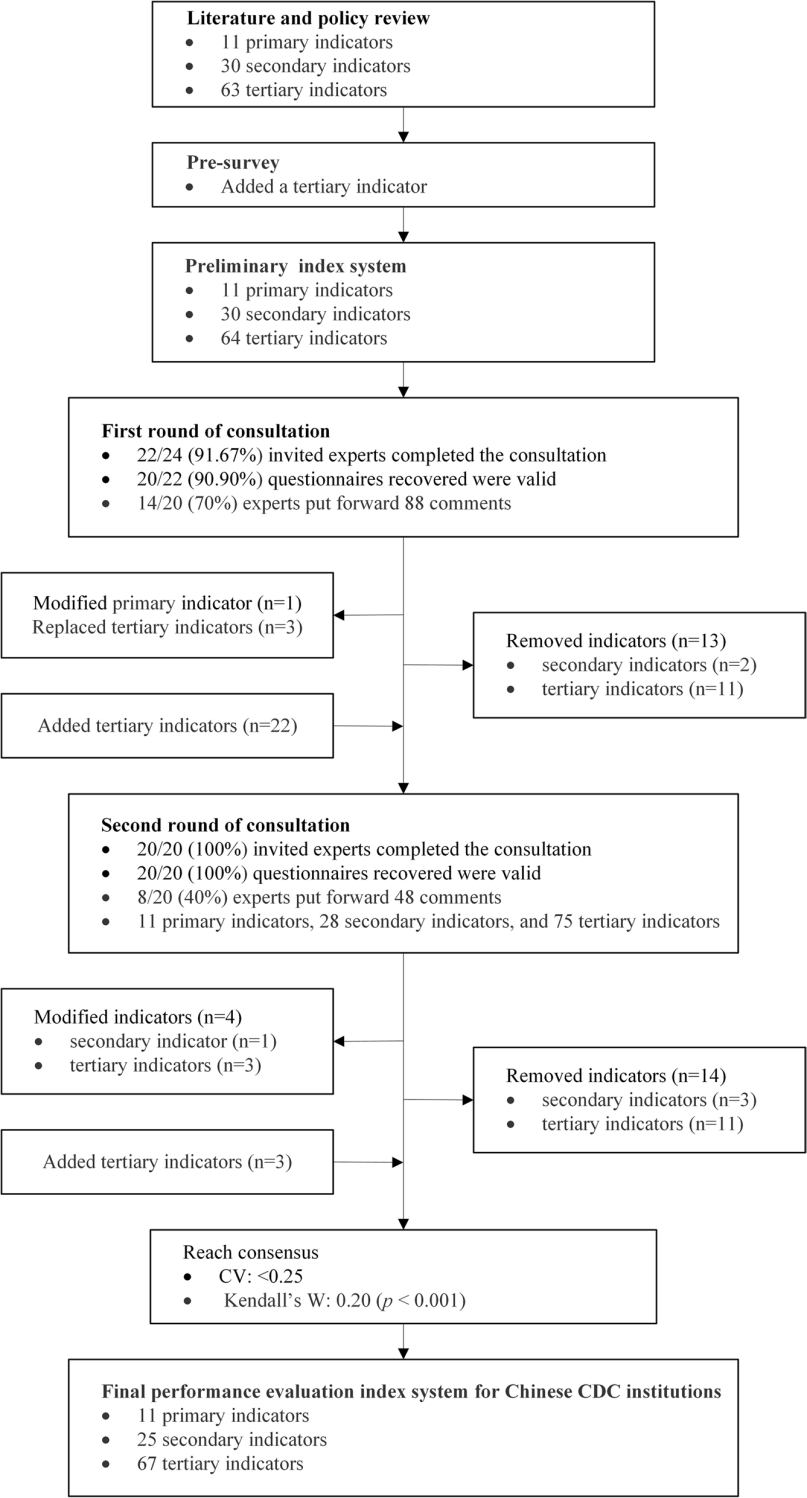
The implementation process of the Delphi method in this study. CV, coefficient of variation; Kendall’s W, Kendall’s coefficient of concordance W; CDC, Centers for Disease Control and Prevention

The Delphi method, developed by the RAND Corporation in 1946, is a widely utilized forecasting approach in diverse fields. It is essentially an anonymous feedback method that seeks expert opinions on a specific issue. After several rounds of feedback, experts’ opinions gradually converge, leading to a more consistent collective judgment [28]. In this study, a pre-survey of experts was conducted on a small scale before the formal Delphi expert consultation to refine the questionnaire. In the initial round of the formal consultation, all experts were presented with the preliminary evaluation index system and background materials. They were requested to assess each indicator across nine dimensions of importance, sensitivity, and accessibility at provincial, city, and county levels, using a 5-point Likert scale. Additionally, experts were asked to self-assess their judgment basis and familiarity and to provide comments and suggestions on the index system. Once the questionnaire was completed, the experts fed the questionnaire back to the research team. After collecting the initial round of questionnaires, the research team analyzed the feedback and developed the second round of questionnaire based on statistical data and expert comments. In the subsequent consultation, experts were presented with the revised index system and selective statistical outcomes from the first round for reference, and were asked to reassess the indicators. If the experts’ opinions were consistent and reliable, the performance evaluation index system for Chinese CDC institutions could be established (Fig. 1).

### Inclusion of experts

At present, there is no exact standard for the selection of experts in the Delphi method, but it is generally believed that the research subjects should have received rigorous training and appropriate skills in the professional field related to the target problem [29]. In 1975, Delbecq, Van de Ven, and Gustafson jointly proposed three suggestions for selecting Delphi research subjects: (1) the senior management decision-makers who will use the results of the Delphi research, (2) the professional staff and their support teams, and (3) the respondents to Delphi questionnaires whose opinions are being sought [30]. Therefore, we correspondingly selected these three types of personnel - government commissioners, university researchers and academic groups, and CDC staff - as the experts to be consulted. Among them, the experts from CDC were required to cover all levels of CDC, including China CDC, provincial CDC, city CDC, and county CDC, and the number of experts from different levels should be balanced as much as possible. To ensure the experts’ representation and authority, we established specific inclusion criteria for experts from various work units and set corresponding requirements for the number of experts based on considerations of convenience and feasibility (Table 1). The final number of experts was 24.

**Table 1:** Expert inclusion criteria and number requirements

**Work unit**	**Inclusion criteria**	**Number of experts**
Centers for Disease Control and Prevention (CDC)	(1) Should have engaged in relevant work at the CDC department for over 5 years. (2) Should have experience serving as a senior leader in the CDC.	10–15
Government departments	(1) At least 5 years of work experience related to public health, market supervision, integrated management. (2) Should have experience serving as a senior leader in government departments.	1–3
Universities or academic groups	(1) The main research direction should focus on social policies, macro health policies, infectious disease surveillance and early warning, or emergency response to public health emergencies, with systematic research work and recognized high-quality research results. (2) It is required to have a good academic reputation.	6–8

### Questionnaire structure and distribution

Based on the preliminary evaluation index system, the research team developed an expert consultation questionnaire. The questionnaire consisted of four parts: (1) Expert profile: including experts’ age, gender, work unit, title, specialty, and years of work experience, (2) Indicator scoring: using a 5-point scale to rate the importance, sensitivity, and accessibility of each indicator. Specifically, very important (5 points), important (4 points), moderate (3 points), unimportant (2 points), very unimportant (1 point); very sensitive (5 points), sensitive (4 points), moderate (3 points), not sensitive (2 points), very insensitive (1 point); very easy to access (5 points), easy to access (4 points), moderate (3 points), not easy to access (2 points), and very difficult to access (1 point). The questionnaire also included a column for modifications to allow experts to make constructive comments on the indicators, (3) Open-ended question: inviting recommendations on the current index system and CDC institutions’ performance evaluation, and (4) Self-assessment: evaluating familiarity with the indicators and the judgment basis. The questionnaire was emailed to the experts, who likewise emailed the research team with feedback on the questionnaire and other relevant questions after completing the questionnaire. The questionnaire data was entered using Excel 2013 software and cross-checked by two researchers.

### Statistical analysis

The reliability of the questionnaire was assessed using Cronbach’s coefficient alpha (Cronbach’s α), which ranges from 0 to 1. It is generally required that Cronbach’s α should be greater than 0.7. The Cronbach’s α for the first round of the expert consultation questionnaire was 0.995, and for the second round, it was 0.996. Both were greater than 0.7, indicating a high overall reliability of the questionnaires. A descriptive analysis of experts’ basic profiles was conducted to demonstrate the professional level of the experts and the richness of their knowledge and work experience related to disease prevention and control. The positivity of experts includes the expert positivity coefficient and the proportion of experts providing comments. The positive coefficient is the responsive rate of the questionnaire, which reflects the level of cooperation of the experts concerning the study. It is generally considered that a responsive rate greater than 70% indicates a high level of positivity from the experts [31]. The proportion of experts providing comments also reflects the experts’ degree of motivation. It refers to the proportion of experts who provide comments and advice among all experts. The authority level of experts is represented by the authority coefficient (Cr). Cr equals the arithmetic mean of judgment basis (Ca) and experts’ familiarity with the indicators (Cs), which mainly depends on self-assessment. Cr greater than 0.50 indicates a relatively good authority level, and greater than 0.70 indicates a high authority level. The quantitative assessment criteria for Ca and Cs are detailed in Table 2. The coordination of expert opinions involves the coefficient of variation (CV) and Kendall’s coefficient of concordance W (Kendall’s W). The CV reflects the degree of coordination in the evaluation of a specific indicator, with a smaller value indicating less divergence among the experts regarding that indicator. It is generally accepted that the CV should be less than 0.25 [31]. Kendall’s W reflects the overall coordination of all experts on all evaluation indicators, with values ranging from 0 to 1 and a more significant value indicating better coordination. The Kendall’s W was tested, with *P* < 0.05 indicating statistical significance. In this study, if the *p*-value of the test for Kendall’s W was less than 0.05 and the CV was less than 0.25, the difference in expert opinions was considered to be within acceptable limits, and consultation was stopped [32].

**Table 2:** Quantitative assessment criteria for judgment basis and familiarity

**Judgment basis**	**Quantitative value**	**Familiarity**	**Quantitative value**
Practical experience	0.8	Familiar	0.8
Theoretical analysis	0.6	Moderate	0.6
Knowledge of peers at home and abroad	0.4	Less familiar	0.4
Intuition	0.2	Unfamiliar	0.2

We selected three measurement scales: full score frequency, arithmetic mean, and CV, with specified corresponding thresholds for each scale. The thresholds for the full score frequency and the arithmetic mean were equal to the mean minus the standard deviation, and indicators with actual scores higher than the thresholds were retained. The threshold for the CV was equal to the mean plus the standard deviation, and indicators with actual scores below the threshold were retained [33, 34]. The screening of indicators was conducted separately at the provincial, city, and county levels. At a certain level, for a certain indicator, if any of its importance, sensitivity, or accessibility did not meet the requirements on all three scales, or if there was a lack of compliance in one or two scales for all of its importance, sensitivity, and accessibility, it was marked as “definite exclusion” (indicated by “ × ”). At a certain level, for a certain indicator, if its importance, sensitivity, and accessibility all met the requirements of the three measurement scales, it was marked as “suggested retention” (indicated by “√”). Other situations were marked as “consideration for exclusion” (indicated by “○”), with experts’ opinions fully considered before decisions were made by the research team.

Data analysis was conducted using SPSS 20.0 statistical software. The mean and standard deviation were calculated for quantitative data that follows a normal distribution, and for qualitative data, the frequency and proportion were calculated.

## Results

### Construction and refinement of the preliminary index system

Considering the diversity of CDC functions, 11 primary indicators were included in the SPO framework. The structural dimension encompassed the integrated support capability. The process dimension encompassed communicable disease prevention and control, chronic non-communicable disease prevention and control, public health emergency response, surveillance, and early warning, health hazard monitoring and control, health education and promotion, information management, technical and skill guidance, and integrated service. The outcome dimension comprised the outcome of disease prevention and control and institutional development and satisfaction evaluation. By referring to previous index systems and reviewing relevant literature, 30 secondary indicators and 63 tertiary indicators were collected. Two experts were invited to conduct a pre-survey to evaluate the content and structure of the questionnaire. Based on the results of the pre-survey and the feedback from the experts, we retained the overall structure of the questionnaire and added a tertiary indicator, “Coverage of full electronic vaccine tracing”. Therefore, the finalized preliminary evaluation index system consisted of 11 primary indicators, 30 secondary indicators, and 64 tertiary indicators.

### Description of expert profile

Based on the expert inclusion criteria, 24 experts were included in the first round, with 20 experts continuing to participate in the second round of consultation. The experts who completed the consultation came from representative CDC institutions, universities, research institutions, and government agencies nationwide. The number of experts from China CDC, provincial CDC, city CDC, and county CDC did not vary much to ensure the representativeness of the index system at different levels of CDC. Their research or work fields covered public health, health management, disease prevention and control, immunization planning, epidemiology, nutrition, and food hygiene. The ages of the experts ranged from 40 to 69 years (first round: 55.8 years ± 7.3 years; second round: 54.9 years ± 6.5 years). The basic characteristics of the experts in the valid questionnaires are detailed in Table 3.

**Table 3:** Basic information on experts

**Classification**	**First round**		**Second round**	
	**Number of people**	**Proportion (%)**	**Number of people**	**Proportion (%)**
**Gender**				
Male	16	80	15	75
Female	4	20	5	25
**Education**				
Undergraduate degree	15	75	17	85
Graduate degree	5	25	3	15
**Work unit**				
University or academic group	7	35	6	30
Government department	2	10	3	15
China CDC	2	10	2	10
Provincial CDC	3	15	4	20
City CDC	3	15	3	15
County CDC	3	15	2	10
**Years of work experience**				
≥ 30 years	11	55	10	50
20–29 years	7	35	8	40
10–19 years	2	10	2	10
**Title**				
Senior	18	90	18	90
Deputy senior	2	10	2	10

*CDC* Centers for Disease Control and Prevention

### Positivity of experts

In the first round of consultation, the expert positivity coefficient was 91.67% (22/24), and 70% (14/20) of experts put forward 88 comments altogether. In the second round, the positivity coefficient was 100% (20/20), and 40% (8/20) of experts made a total of 48 comments (Table 4). These results showed that most experts cared about the study and were highly motivated to participate.

**Table 4:** Recovery of questionnaires and positivity of the experts

**Item**	**First round**	**Second round**
Number of questionnaires distributed	24	20
Number of questionnaires recovered	22	20
Questionnaire responsive rate (%)	91.67	100
Number of valid questionnaires	20	20
Valid questionnaire responsive rate (%)	90.90	100
Number of experts providing comments	14	8
Proportion of experts providing comments (%)	70	40
Number of comments	88	48

### Authority of experts

The authority of experts could reflect the reliability of consulting results. The mean values of Ca for the two rounds of consultation were 0.77 and 0.73, respectively. The mean values of Cs for the two rounds were 0.74 and 0.70, respectively. Cr = (Ca + Cs)/2, and Cr for the two rounds were 0.76 and 0.72, respectively, both greater than 0.70. Therefore, the expert authority of this study could be considered high, and the counseling results were credible.

### Coordination of expert opinions

The coordination of expert opinions was measured by Kendall’s W and coefficient of variation. The Kendall’s W for the two rounds were 0.19 and 0.20, respectively, both proven to be significant (*p* < 0.001), indicating the consistency of expert opinions (Table 5). In addition, the CV for the two rounds ranged from 0.13 to 0.32 and from 0.12 to 0.23, respectively (Table 6). After the second round of consultation, the CV was within acceptable limits (< 0.25).

**Table 5:** Kendall’s coefficient of concordance W and its test results

**Statistic**	**First round**	**Second round**
Number of indicators	64	75
Number of experts	20	20
Kendall’s W	0.19	0.20
Chi-square	1603.84	1211.87
*P*-value	< 0.001	< 0.001

Kendall’s W, Kendall’s coefficient of concordance W

**Table 6:** Means and standard deviations of full score frequencies, arithmetic means and coefficients of variation

**Level**	**First round**			**Second round**		
	**Full score frequency**	**Arithmetic mean**	**Coefficient of variation**	**Full score frequency**	**Arithmetic mean**	**Coefficient of variation**
**Provincial level**						
Importance	0.76 ± 0.14	4.66 ± 0.23	0.13 ± 0.06	0.76 ± 0.12	4.69 ± 0.18	0.12 ± 0.06
Sensitivity	0.54 ± 0.11	4.26 ± 0.23	0.22 ± 0.05	0.53 ± 0.12	4.23 ± 0.21	0.23 ± 0.04
Accessibility	0.55 ± 0.14	4.30 ± 0.26	0.20 ± 0.06	0.58 ± 0.14	4.36 ± 0.26	0.19 ± 0.05
**City level**						
Importance	0.71 ± 0.15	4.62 ± 0.23	0.14 ± 0.05	0.73 ± 0.11	4.66 ± 0.18	0.13 ± 0.05
Sensitivity	0.49 ± 0.11	4.17 ± 0.25	0.24 ± 0.05	0.50 ± 0.11	4.21 ± 0.19	0.23 ± 0.04
Accessibility	0.50 ± 0.15	4.13 ± 0.26	0.24 ± 0.05	0.54 ± 0.14	4.31 ± 0.24	0.20 ± 0.04
**County level**						
Importance	0.67 ± 0.15	4.52 ± 0.26	0.17 ± 0.06	0.72 ± 0.13	4.63 ± 0.19	0.14 ± 0.05
Sensitivity	0.45 ± 0.14	4.02 ± 0.31	0.27 ± 0.05	0.48 ± 0.13	4.16 ± 0.23	0.23 ± 0.04
Accessibility	0.47 ± 0.16	3.94 ± 0.33	0.32 ± 0.06	0.48 ± 0.13	4.14 ± 0.25	0.23 ± 0.05

### Indicator screening

Based on the results of the expert consultation, full score frequencies, arithmetic means, and CV of importance, sensitivity, and accessibility were calculated for all indicators at the provincial, city, and county levels. The full score frequency ranges for the two rounds were 0.45 to 0.75 and 0.48 to 0.76, respectively; the arithmetic mean ranges were 3.94 to 4.66 and 4.14 to 4.69, respectively; and the CV ranges were 0.13 to 0.32 and 0.12 to 0.23, respectively (Table 6). Thresholds for the full score frequency, arithmetic mean, and CV were then calculated. The actual results for each indicator were compared with the thresholds of the three measurement scales. Following the established rules, the indicators were screened at the provincial, city, and county levels, and the subjective opinions of experts were taken into account in the process.

After the first round of expert consultation, “D. Public health emergency response, surveillance, and early warning” was changed to “D. Public health emergency response”. “B12. Misreporting rate of infectious diseases in medical institutions” was replaced with “B12. Statutory infectious disease reporting rate in medical institutions”; “B112. Monitoring of unexplained pneumonia and human avian influenza” was replaced with “B112. Severe respiratory syndrome monitoring”; and “E12. Completion rate of occupational disease reporting” was replaced with “E12. Occupational health monitoring rate”. Two secondary and 11 tertiary indicators were removed, and the application scope of nine tertiary indicators was adjusted. In addition, 22 tertiary indicators were added in accordance with the experts’ suggestions, as follows: “Growth rate of annual government funding” under “A1. Fund”, “Proportion of professional technicians”, “Proportion of staffing”, and “Proportion of senior positions and highly educated technicians” under “A2. Talent team construction”, “Class III biosafety lab” under “A4. Laboratory capacity and safety”, “Monitoring completion rate for vaccine-preventable infectious diseases”, “Coverage rate of standardized vaccination clinics”, and “Completion rate of population antibody level monitoring” under “B6. Immunization planning and vaccine management”, “Target achievement rate of the National Healthy Lifestyle” under “C1. Overview of chronic non-communicable disease prevention and control”, “Emergency stockpile completeness rate” under “D1. Emergency disposal”, “Air quality monitoring rate” under “E3. Environmental hazards control”, “Awareness rate of key hygiene and disease prevention knowledge among target groups” and “Awareness rate of blood pressure and blood glucose in the population” under “F2. Health education for target groups”, “Comprehensive evaluation of abilities and qualifications” and “Project budget completion rate” under “I1. Other work capacities”, “Compliance rate for communicable diseases under planning control”, “Total incidence of infectious diseases”, and “Mortality rate for statutory infectious diseases” under “J1. Outcome indicators for communicable disease prevention and control”, “Annual growth rate of health literacy” and “Formation rate of basic hygiene and disease prevention behaviors of the population” under “J2. Outcome indicators for chronic non-communicable disease prevention and control”, and “Public satisfaction” under “K3. Satisfaction evaluation” (Additional file 1). Following the first round of indicator screening, the index system for the second round of expert consultation comprised 11 primary indicators, 28 secondary indicators, and 75 tertiary indicators.

After the second round of expert consultation, “B3. Prevention and control of tuberculosis and leprosy” was changed to “B3. Tuberculosis prevention and control”. “A24. Proportion of senior positions and highly educated technicians” was amended to “A24. Proportion of highly educated technicians”; “B13. Infectious disease surveillance completion rate” was amended to “B13. Infectious disease surveillance integrity rate”; and “B18. Priority infectious disease surveillance completion rate” was amended to “B18. Priority infectious disease surveillance integrity rate”. Three secondary and 11 tertiary indicators were removed, and the application scope of 12 tertiary indicators was adjusted. In addition, three tertiary indicators were added following the experts’ suggestions, as follows: “Accuracy of syphilis epidemic reporting” and “Consultation and testing rate for syphilis in high-risk populations” under “B2. AIDS and syphilis prevention and control” and “Per capita days of professional guidance at the grassroots” under “H2. Technical guidance” (Additional file 1). Due to the good consistency of expert opinions in the second round of consultation, the consultation outcomes were desirable, and there was no need for a subsequent round of consultation. The final performance evaluation index system for Chinese CDC institutions comprised 11 primary, 25 secondary, and 67 tertiary indicators (Table 7).

**Table 7:** Performance evaluation index system for CDC institutions in Chinese provinces, cities, and counties

Evaluation indicators	Scope of application		
	**Province**	**City**	**County**
A. Integrated support capability	√	√	√
A1. Fund	√	√	√
A11. Proportion of fiscal appropriations to annual expenditures	√	√	√
A12. Growth rate of annual government funding	√	√	√
A2. Talent team construction	√	√	√
A21. Proportion of healthcare technicians	√	√	√
A22. Proportion of professional technicians	√	√	√
A23. Proportion of highly educated technicians	√	√	
A3. Infrastructure, materials, and equipment	√	√	√
A31. Compliance rate of inspection equipment	√	√	√
A32. Informatization construction evaluation index	√		
A4. Laboratory capacity and safety	√	√	√
A41. Implementation rate of laboratory testing programs	√	√	√
A42. Laboratory safety management	√	√	√
A43. Laboratory quality control coverage	√	√	√
A44. Class III biosafety lab	√		
A5. Party construction	√	√	√
A51. Implementation of the party construction work responsibility system	√	√	√
B. Communicable disease prevention and control	√	√	√
B1. Overview of infectious disease prevention and control	√	√	√
B11. Comprehensive evaluation rate of information quality in epidemic reporting	√	√	√
B12. Statutory infectious disease reporting rate in medical institutions		√	√
B13. Infectious disease surveillance integrity rate	√	√	√
B14. Outbreak standardized disposal index		√	√
B15. Coverage rate of direct network reporting of infectious diseases		√	√
B16. Timely response rate of automatic warning signals for infectious diseases	√	√	√
B17. Priority infectious disease surveillance integrity rate	√	√	
B18. Experimental diagnosis rate of priority infectious diseases	√	√	√
B19. Laboratory testing capacity for priority infectious diseases	√	√	√
B110. Incidence of AFP cases in children under 15 years old	√	√	√
B111. Severe respiratory syndrome monitoring		√	√
B2. AIDS and syphilis prevention and control		√	√
B21. Coverage of interventions for high-risk groups of AIDS		√	√
B22. Proportion of HIV-infected and AIDS patients followed up with interventions		√	√
B23. Accuracy of syphilis epidemic reporting		√	√
B24. Consultation and testing rate for syphilis in high-risk populations		√	√
B3. Tuberculosis prevention and control	√	√	√
B31. Incidence of tuberculosis	√	√	√
B32. Management rate of tuberculosis patients	√	√	√
B33. Supervisory coverage of tuberculosis control		√	√
B4. Surveillance of insect-borne infectious diseases	√	√	√
B41. Completion rate of vector monitoring	√	√	√
B5. Endemic disease	√	√	√
B51. Completion rate of endemic disease monitoring	√	√	√
B6. Immunization planning and vaccine management	√	√	√
B61. Childhood vaccination rate	√	√	√
B62. Standardized treatment rate of suspected abnormal reactions to vaccination	√	√	√
B63. Children’s vaccination certification rate		√	√
B64. Coverage of full electronic vaccine tracing	√	√	√
B65. Monitoring completion rate for vaccine-preventable infectious diseases	√	√	√
B66. Coverage rate of standardized vaccination clinics	√	√	√
C. Chronic non-communicable disease prevention and control	√	√	√
C1. Overview of chronic non-communicable disease prevention and control	√	√	√
C11. Coverage rate of whole-population cause-of-death monitoring	√	√	
C12. Standardized registration and reporting rate of causes of death	√	√	√
C13. Target achievement rate of the National Healthy Lifestyle			√
C2. Monitoring of risk factors for chronic non-communicable diseases	√	√	
C21. Coverage rate of monitoring chronic disease risk factors	√	√	
D. Public health emergency response	√	√	√
D1. Emergency disposal	√	√	√
D11. Regulated disposal index	√	√	√
D12. Timely incident reporting rate	√	√	√
D13. Information direct network reporting rate	√	√	√
D14. Emergency stockpile completeness rate	√	√	√
E. Health hazard monitoring and control	√	√	√
E1. Monitoring and control of occupational disease hazards	√	√	√
E11. Completion rate of priority occupational disease monitoring	√	√	√
E12. Occupational health monitoring rate	√	√	√
E2. Foodborne disease prevention and control	√	√	√
E21. Completion rate of food safety risk monitoring	√	√	√
E3. Environmental hazards control	√	√	√
E31. Drinking water monitoring rate	√	√	√
F. Health education and promotion		√	√
F1. Health education for target groups		√	√
F11. Behavioral intervention index for target groups			√
F12. Awareness rate of key hygiene and disease prevention knowledge among target groups		√	√
F13. Awareness rate of blood pressure and blood glucose in the population		√	√
G. Information management	√	√	
G1. Information utilization and analysis	√	√	
G11. Evaluation index for data analysis	√	√	
H. Skill and technical guidance	√	√	√
H1. Skill training	√	√	√
H11. Job skill training rate	√	√	√
H2. Technical guidance	√	√	√
H21. Coverage rate of grassroots professional guidance	√	√	√
H22. Per capita days of professional guidance at the grassroots	√	√	√
I. Integrated service indicators	√	√	√
I1. Other work capacities	√	√	√
I11. Completion rate of directive work	√	√	√
I12. Comprehensive evaluation of abilities and qualifications	√	√	√
I13. Project budget completion rate	√	√	√
J. Outcome of disease prevention and control	√	√	√
J1. Outcome indicators for communicable disease prevention and control	√	√	√
J11. Disability rate in newly discovered leprosy patients	√	√	√
J12. Achievement rate of parasitic disease prevention and control goals	√	√	√
J13. Compliance rate for communicable diseases under planning control	√	√	√
J14. Mortality rate for statutory infectious diseases	√	√	√
K. Institutional development and satisfaction evaluation	√	√	√
K1. Scientific research capacity	√	√	
K11. Comprehensive evaluation of scientific research projects	√	√	
K2. Satisfaction evaluation	√	√	√
K21. Employee satisfaction	√	√	√
K22. Public satisfaction	√	√	√

*CDC* Centers for Disease Control and Prevention, *AFP* Acute flaccid paralysis

## Discussion

### Principal findings of this study

After expert consultation, this study ultimately developed a new performance evaluation index system for Chinese CDC institutions at the provincial, city, and county levels, as follows: ten primary indicators, 23 secondary indicators, and 53 tertiary indicators for provincial CDC; 11 primary indicators, 25 secondary indicators; 63 tertiary indicators for city CDC; and ten primary indicators, 22 secondary indicators, and 59 tertiary indicators for county CDC (Table 7). These indicators were formulated through Delphi expert consultation, integrating professional insights and actual requirements, ensuring scientific rigor and practical relevance.

The Delphi consulting process involved experts from diverse work units, including CDC at all levels, government departments, and academic institutions, creating a well-structured and multidisciplinary expert composition. The experts came from the eastern, central, and western regions of China, covering different geographic areas of disease prevention and control, and were able to provide comprehensive and representative opinions. Besides, unlike most previous expert consultations, this study evaluated not only the importance but also the sensitivity and accessibility of the indicators. In the two rounds of expert group scoring, the average score for indicator importance was above 4.5 out of 5, and the average scores for sensitivity and accessibility were nearly all above 4.0. The scoring results showed that the indicators in the index system were practical and accurately reflected the characteristics of CDC responsibilities, which contributed to the problem-solving and optimization of the institutions. Moreover, considering the variations in responsibilities and work priorities across different levels of CDC institutions, the performance evaluation index systems were tailored for provincial, municipal, and county levels. This differentiation allowed for a more precise assessment of institutions at different levels in diverse areas, thus offering a scientific foundation for management decisions.

### Interpretation of results

In this study, two rounds of the Delphi method were carried out. How many rounds of discussion does the Delphi method require? Theoretically, the answer is that until a consensus is reached. However, as the number of Delphi rounds gradually increases, the loss of energy and attention from the research subjects becomes greater. Too many rounds may result in participants agreeing to certain viewpoints just to get through as quickly as possible, which may lead to a false consensus [35]. There is research suggesting that the process should stop when the difference in responses decreases to a certain level, but the specific criteria remain uncertain [28, 36, 37]. Therefore, based on predetermined consistency criteria in this study, we stopped the consultation when the *p*-value of the test for Kendall’s W was less than 0.05, and the CV was less than 0.25 in the second round of consultation. According to the results of relevant literature reviews, most Delphi methods are completed within 2–3 rounds [28, 36, 38], and the rounds of this study were basically consistent with previous studies.

The Kendall’s W in this study was 0.19 and 0.20 in two rounds of consultation, respectively. For general assessment criteria, these results were far from 1, indicating that the consistency of the experts in this study may be poor. However, Kendall’s W can be used directly to determine the level of consistency when the number of experts is ≤ 20 and the number of indicators is ≤ 7. If this condition is not met, the chi-square test should be used to test for significance [39]. As the number of evaluation indicators in this study far exceeded 7 (first round: 64, second round: 75), the chi-square statistic was used to test for consistency, and the results were significant (*p* < 0.001). In addition, in many studies with a large number of evaluation indicators, the Kendall’s W was low (0.10–0.31), but the chi-squared test showed statistically significant differences (*p* < 0.001) [40–42]. Therefore, the Kendall’s W and its test results in this study were consistent with previous studies.

Compared to the 2015 index system [6], this study refined the primary and secondary indicators. For the primary indicators, the study classified the performance evaluation into 11 aspects rather than generalizing them into “Social benefits”, “Service delivery”, “Integrated management”, and “Sustainable development”, as in the 2015 index system. For the secondary indicators, for example, “Infectious disease prevention and control” was further refined into “Overview of infectious disease prevention and control”, “AIDS and syphilis prevention and control”, “Tuberculosis prevention and control”, “Surveillance of insect-borne infectious diseases”, “Endemic disease”, and “Immunization planning and vaccine management”. The refinement of the indicators helped enhance the relevance and practicality of the evaluation system and contributed to developing more precise assessments and improvement actions for problems in different areas rather than just general guidelines. Additionally, this study has made a more reasonable classification of the tertiary indicators, such as the “Job skill training rate”, which was originally part of “Talent team construction”, and was classified as “Skill training”, thus ensuring the accuracy of the assessment and avoiding unnecessary overlap and confusion.

This study streamlined certain indicators, which were not aligned with the evolving functions of CDC. Indicators like the “Qualification rate of continuing medical education”, “Number of major health promotion activities”, “Completion rate of occupational disease reporting”, and “Coverage rate of disinfection quality monitoring” were excluded. As a result, the developed index system was more aligned with the actual situation of CDC’s work. With references from international experiences, this study introduced indicators to evaluate funding and talent team building. The “Proportion of fiscal appropriations to annual expenditures” and “Growth rate of annual government funding” were designed to assess the input and utilization of the fiscal budget. “Proportion of professional technicians” and “Proportion of highly educated technicians” were aimed at evaluating the capacity of professional talents in CDC. By strengthening the requirements for financial and human resources, the overall protection capacity of the organization would be strengthened. Furthermore, this study responded to the national policy by adding relevant indicators [17, 20, 25]. For example, “Timely response rate of automatic warning signals for infectious diseases”, “Laboratory testing capacity for priority infectious diseases”, “Severe respiratory syndrome monitoring”, and “Emergency stockpile completeness rate” aimed to encourage CDC to strengthen their capabilities in surveillance and early warning of emerging infectious disease outbreaks, as well as responsiveness to public health emergencies through performance evaluation. The “Coverage of full electronic vaccine tracing” and “Coverage rate of standardized vaccination clinics” were introduced to reinforce vaccine management and standardize preventive immunization.

### Possible applications

On the theoretical side, conducting scientific performance evaluation is one of the most crucial means of disease prevention and control. By developing assessment indicators and evaluation systems, it is possible to quantify and measure the performance of CDC institutions. It can also provide valuable references for policymakers and managers, allowing them to understand institutional responsibilities, identify problems, and formulate improvement measures. Moreover, it boosts organization members’ motivation to enhance their work quality and efficiency and fosters continuous improvement and development of the disease prevention and control system [43, 44]. This study’s findings enriched performance evaluation theories for CDC institutions, offering essential guidance for institutional improvement and optimization.

On the practical side, the current CDC system in China is in a critical period of reform, and the previous performance evaluation index systems are no longer suitable for the current state of health development [3]. The index system constructed in this study systematically and scientifically evaluated the CDC institutions’ work capabilities in various aspects under the framework of service resources, service process, and service results. In addition, since this study screened the indicators at the provincial, city, and county levels, the final index system was applicable and operable at different levels of CDC agencies. Overall, the performance evaluation index system developed in this study has provided an effective evaluation tool for CDC to enhance their work quality.

### Limitations of the study

First, despite specific criteria and strict steps in the expert consultation process, the Delphi method unavoidably introduced some subjectivity, which was the primary source of potential bias. In the subsequent evaluation practice, CDC organizations can assess the indicators based on their circumstances to evaluate the index system’s applicability in a more scientific way. Second, due to time and labor constraints, we selected only 24 experts for the Delphi survey. The sample size of the expert panel in this study was relatively small, but it was consistent with the number of experts in many previous studies [29, 33, 45, 46]. It is important to note that a larger panel size may result in greater rounds needed to achieve consensus. Third, due to the busy schedules of the experts, experts were unable to participate in further studies, and we could not implement the analytical hierarchy process to differentiate the weights of each indicator. Therefore, we initially used balanced weights, which may not be scientifically sound. Last, the index system developed in this study was based on China’s health situation and policies, and all the experts consulted were from China. Consequently, the index system may not be directly applicable to other countries, but it could offer valuable insights for the performance evaluation of CDC in other nations.

Given these limitations, if the conditions were mature, the scope and scale of the Delphi expert consultation could be expanded in future studies to enhance the representativeness of the findings. In future practical applications, the weights of the indicators will be changed promptly according to the feedback of the survey data through objective weighting methods such as the entropy value method or principal component analysis. Simultaneously, the index system should be dynamically adjusted according to the actual circumstances and health policies so as to further improve the assessment standards and facilitate the high-quality development of Chinese disease prevention and control system.

## Conclusions

Based on the current health situation and national policies, the performance evaluation index system we developed for Chinese CDC institutions through the Delphi method was highly authoritative, scientific, and feasible. This index system holds great importance as it provides an evaluation tool for CDC work and serves as a crucial reference for institutional optimization. During its subsequent dissemination and usage, the index system should be dynamically adjusted according to the actual circumstances and health policies, so as to further improve the assessment standards and facilitate the high-quality development of Chinese disease prevention and control system.

### Supplementary Information

Supplementary Material 1.

## Data Availability

The data that support the findings of this study are not openly available in order to protect the privacy of the expert panel, but deidentification data could be made available from the corresponding author upon reasonable request.

## Abbreviations

CDC: Centers for Disease Control and Prevention
SPO: Structure-Process-Outcome
Cronbach’s α: Cronbach’s coefficient alpha
Cr: Authority coefficient
Ca: Judgment basis
Cs: Familiarity coefficient
CV: Coefficient of variation
Kendall’s W: Kendall’s coefficient of concordance W
