# Supplementary material for: The development of a performance evaluation index system for Chinese Centers for Disease Control and Prevention: a Delphi consensus study
Source: Glob Health Res Policy. 2024 Jul 23;9:28. doi: 10.1186/s41256-024-00367-w (PMC11265441; doi:10.1186/s41256-024-00367-w)
Supplement: Supplementary file 1 — Supplementary Material 1. [file 41256_2024_367_MOESM1_ESM.docx]

**Additional file 1**

**Table 1** The results of the first round of expert consultation on indicator screening

| **Indicator** | **Screening based on scoring** | | | **Comment** | **Decision** |
| --- | --- | --- | --- | --- | --- |
|  | **Province** | **City** | **County** |  |  |
| A. Integrated support capability | ‒ | ‒ | ‒ | Add tertiary indicators | Retain and add subordinate tertiary indicators |
| A1. Fund | ‒ | ‒ | ‒ | NA | Retain |
| A11. Proportion of fiscal appropriations to annual expenditures | √ | √ | √ | NA | Retain |
| A12. Project budget completion rate | √ | √ | √ | NA | Retain |
| A2. Talent team construction | ‒ | ‒ | ‒ | NA | Retain |
| A21. Proportion of healthcare technicians | √ | √ | √ | Modify indicator evaluation criteria | Retain and modify indicator evaluation criteria |
| A3. Infrastructure, materials, and equipment | ‒ | ‒ | ‒ | NA | Retain |
| A31. Compliance rate of inspection equipment | √ | √ | √ | Remove or modify indicator evaluation criteria | Retain and modify indicator evaluation criteria |
| A32. Instrumentation uptime rate | √ | √ | √ | Modify indicator evaluation criteria | Retain and modify indicator evaluation criteria |
| A33. Informatization construction evaluation index | ○ | √ | ○ | NA | Retain |
| A4. Laboratory capacity and safety | ‒ | ‒ | ‒ | NA | Retain |
| A41. Implementation rate of laboratory testing programs | √ | √ | √ | NA | Retain |
| A42. Laboratory safety management | √ | √ | √ | NA | Retain |
| A43. Laboratory quality control coverage | √ | √ | √ | NA | Retain |
| A5. Party construction | ‒ | ‒ | ‒ | NA | Retain |
| A51. Implementation of the party construction work responsibility system | √ | √ | √ | NA | Retain |
| B. Communicable disease prevention and control | ‒ | ‒ | ‒ | NA | Retain |
| B1. Overview of infectious disease prevention and control | ‒ | ‒ | ‒ | Distinguish applicable indicators for provinces, cities, and counties | Retain |
| B11. Comprehensive evaluation rate of information quality in epidemic reporting | √ | √ | √ | Modify indicator evaluation criteria | Retain and modify indicator evaluation criteria |
| B12. Misreporting rate of infectious diseases in medical institutions | × | ○ | ○ | Replace the indicator | Replace the indicator and remove at the provincial level |
| B13. Infectious disease surveillance completion rate | √ | √ | √ | Modify indicator evaluation criteria | Retain |
| B14. Outbreak standardized disposal index | ○ | ○ | √ | Modify indicator evaluation criteria | Retain and modify indicator evaluation criteria |
| B15. Coverage rate of direct network reporting of infectious diseases | √ | √ | √ | Suggested removal | Retain |
| B16. Reported incidence of class A and B statutory infectious diseases | ○ | ○ | √ | Remove | Remove at the provincial and city levels |
| B17. Timely response rate of automatic warning signals for infectious diseases | ○ | √ | √ | Modify indicator evaluation criteria | Retain and modify indicator evaluation criteria |
| B18. Priority infectious disease surveillance completion rate | √ | √ | √ | Modify indicator evaluation criteria and remove at the country level | Remove at the country level |
| B19. Experimental diagnosis rate of priority infectious diseases | √ | ○ | ○ | Modify indicator evaluation criteria | Retain |
| B110. Laboratory testing capacity for priority infectious diseases | √ | √ | ○ | NA | Retain |
| B111. Incidence of AFP cases in children under 15 years old | √ | √ | √ | NA | Retain and modify indicator evaluation criteria |
| B112. Monitoring of unexplained pneumonia and human avian influenza | √ | √ | √ | Replace the indicator | Replace the indicator |
| B2. AIDS and syphilis prevention and control | ‒ | ‒ | ‒ | NA | Retain |
| B21. Coverage of interventions for high-risk groups of AIDS | ○ | √ | √ | NA | Retain |
| B22. Proportion of HIV-infected and AIDS patients followed up with interventions | √ | ○ | √ | NA | Retain |
| B23. Proportion of HIV-infected and AIDS patients receiving antiviral treatment | √ | √ | √ | Remove | Remove |
| B3. Prevention and control of tuberculosis and leprosy | ‒ | ‒ | ‒ | NA | Retain |
| B31. Incidence of tuberculosis | √ | √ | √ | NA | Retain |
| B32. Management rate of tuberculosis patients | √ | √ | √ | NA | Retain |
| B33. Supervisory coverage of tuberculosis control | ○ | √ | √ | Remove at the provincial level | Remove at the provincial level |
| B4. Surveillance of insect-borne infectious diseases | ‒ | ‒ | ‒ | NA | Retain |
| B41. Completion rate of vector monitoring | √ | √ | √ | NA | Retain |
| B5. Endemic disease | ‒ | ‒ | ‒ | NA | Retain |
| B51. Completion rate of endemic disease monitoring | ○ | √ | √ | NA | Retain |
| B6. Immunization planning and vaccine management | ‒ | ‒ | ‒ | Add tertiary indicators | Retain and add subordinate tertiary indicators |
| B61. Childhood vaccination rate | √ | √ | √ | Modify indicator evaluation criteria | Retain and modify indicator evaluation criteria |
| B62. Standardized treatment rate of suspected abnormal reactions to vaccination | √ | √ | √ | NA | Retain |
| B63. Children’s vaccination certification rate | ○ | √ | √ | Modify indicator evaluation criteria | Retain and modify indicator evaluation criteria |
| B64. Coverage of full electronic vaccine tracing | √ | √ | √ | NA | Retain |
| C. Chronic non-communicable disease prevention and control | ‒ | ‒ | ‒ | NA | Retain |
| C1. Overview of chronic non-communicable disease prevention and control | ‒ | ‒ | ‒ | Add tertiary indicators | Retain and add subordinate tertiary indicators |
| C11. Coverage rate of whole-population cause-of-death monitoring | √ | √ | × | Modify indicator evaluation criteria | Modify indicator evaluation criteria and remove at the country level |
| C12. Standardized registration and reporting rate of causes of death | ○ | ○ | √ | NA | Retain |
| C13. Chronic non-communicable disease management rate | × | × | × | Suggested removal | Remove |
| C14. Filing rate of residents’ health records (electronic or paper) | × | × | × | Suggested removal | Remove |
| C15. Health records coverage rate of chronic disease patients | × | × | ○ | Suggested removal | Remove |
| C2. Monitoring of risk factors for chronic non-communicable diseases | ‒ | ‒ | ‒ | NA | Retain |
| C21. Coverage rate of monitoring chronic disease risk factors | √ | √ | × | Remove or modify indicator evaluation criteria | Modify indicator evaluation criteria and remove at the country level |
| C22. Nutrition monitoring coverage rate | × | × | × | Remove at the country level | Remove |
| D. Public health emergency response, surveillance, and early warning | ‒ | ‒ | ‒ | Modify the name | Modify the name |
| D1. Emergency disposal | ‒ | ‒ | ‒ | Add tertiary indicators | Retain and add subordinate tertiary indicators |
| D11. Regulated disposal index | ○ | ○ | ○ | Modify indicator evaluation criteria | Retain and modify indicator evaluation criteria |
| D12. Untimely incident reporting rate | √ | √ | √ | Remove | Remove |
| D13. Timely incident reporting rate | √ | √ | √ | Retain | Retain |
| D14. Information direct network reporting rate | ○ | ○ | ○ | Modify indicator evaluation criteria | Retain and modify indicator evaluation criteria |
| E. Health hazard monitoring and control | ‒ | ‒ | ‒ | NA | Retain |
| E1. Monitoring and control of occupational disease hazards | ‒ | ‒ | ‒ | NA | Retain |
| E11. Completion rate of priority occupational disease monitoring | √ | √ | ○ | NA | Retain |
| E12. Completion rate of occupational disease reporting | √ | ○ | ○ | Replace the indicator | Replace the indicator |
| E2. Foodborne disease prevention and control | ‒ | ‒ | ‒ |  | Retain |
| E21. Completion rate of food safety risk monitoring | √ | ○ | ○ | Add tertiary indicators | Retain |
| E3. Environmental hazards control | ‒ | ‒ | ‒ | Add tertiary indicators | Retain and add subordinate tertiary indicators |
| E31. Drinking water monitoring rate | √ | √ | √ | Modify indicator evaluation criteria | Retain and modify indicator evaluation criteria |
| E4. Disinfection quality monitoring | ‒ | ‒ | ‒ | NA | Retain |
| E41. Coverage rate of disinfection quality monitoring | ○ | ○ | ○ | NA | Retain |
| F. Health education and promotion | ‒ | ‒ | ‒ | NA | Retain |
| F1. Public health education | ‒ | ‒ | ‒ | NA | Remove |
| F11. Number of major health promotion activities | × | × | × | NA | Remove |
| F2. Health education for target groups | ‒ | ‒ | ‒ | Add tertiary indicators | Retain and add subordinate tertiary indicators |
| F21. Behavioral intervention index for target groups | ○ | ○ | ○ | NA | Retain |
| G. Information management | ‒ | ‒ | ‒ | NA | Retain |
| G1. Information gathering | ‒ | ‒ | ‒ | NA | Retain |
| G11. Evaluation index for timeliness and completeness of data reporting | ○ | ○ | ○ | NA | Retain |
| G2. Information utilization and analysis | ‒ | ‒ | ‒ | NA | Retain |
| G21. Evaluation index for data analysis | ○ | ○ | × | NA | Remove at the country level |
| H. Skill and technical guidance | ‒ | ‒ | ‒ | Distinguish applicable indicators for provinces, cities, and counties | Retain |
| H1. Skill training | ‒ | ‒ | ‒ | NA | Retain |
| H11. Job skill training rate | √ | √ | √ | Modify indicator evaluation criteria | Retain and modify indicator evaluation criteria |
| H2. Technical guidance | ‒ | ‒ | ‒ | NA | Retain |
| H21. Coverage rate of grassroots professional guidance | ○ | √ | ○ | Modify indicator evaluation criteria | Retain and modify indicator evaluation criteria |
| I. Integrated service indicators | ‒ | ‒ | ‒ | NA | Retain |
| I1. Other work capacities | ‒ | ‒ | ‒ | NA | Retain |
| I11. Completion rate of directive work | √ | √ | √ | Suggested removal | Retain |
| I12. Work program completion rate | × | ○ | √ | Remove | Remove |
| J. Outcome of disease prevention and control | ‒ | ‒ | ‒ | NA | Retain |
| J1. Outcome indicators for communicable disease prevention and control | ‒ | ‒ | ‒ | Add tertiary indicators | Retain and add subordinate tertiary indicators |
| J11. Disability rate in newly discovered leprosy patients | × | × | ○ | NA | Remove at the provincial and city levels |
| J12. Achievement rate of parasitic disease prevention and control goals | ○ | ○ | ○ | NA | Retain |
| J2. Outcome indicators for chronic non-communicable disease prevention and control | ‒ | ‒ | ‒ | NA | Retain |
| J21. Resident awareness rate of health education knowledge | × | × | × | Remove | Remove |
| K. Institutional development and satisfaction evaluation | ‒ | ‒ | ‒ | NA | Retain |
| K1. Continuing medical education | ‒ | ‒ | ‒ | NA | Remove |
| K11. Qualification rate of continuing medical education | × | × | × | Remove | Remove |
| K2. Scientific research capacity | ‒ | ‒ | ‒ | Add tertiary indicators | Retain |
| K21. Comprehensive evaluation of scientific research projects | ○ | × | × | Modify indicator evaluation criteria | Modify indicator evaluation criteria and remove at the city and country levels |
| K3. Satisfaction evaluation | ‒ | ‒ | ‒ | Add tertiary indicators | Retain and add subordinate tertiary indicators |
| K31. Employee satisfaction | √ | ○ | ○ | Modify indicator evaluation criteria | Retain and modify indicator evaluation criteria |

“√” indicates suggested retention, “○” indicates consideration for exclusion, and “×” indicates definite exclusion. “‒” indicates that indicates that primary and secondary indicators were not scored directly, but were adjusted based on the screening of subordinate tertiary indicators and expert comments. NA indicates that the experts did not comment on the corresponding indicators. AFP, acute flaccid paralysis.

**Table 2** The results of the second round of expert consultation on indicator screening

| **Indicator** | **Screening based on scoring** | | | **Comment** | **Decision**  **Province** |
| --- | --- | --- | --- | --- | --- |
|  | **Province** | **City** | **County** |  |  |
| A. Integrated support capability | ‒ | ‒ | ‒ | NA | Retain |
| A1. Fund | ‒ | ‒ | ‒ | NA | Retain |
| A11. Proportion of fiscal appropriations to annual expenditures | √ | √ | √ | Modify indicator evaluation criteria | Retain and modify indicator evaluation criteria |
| A12. Project budget completion rate | √ | √ | √ | Remove | Remove |
| A13. Growth rate of annual government funding | ○ | ○ | ○ | NA | Retain |
| A2. Talent team construction | ‒ | ‒ | ‒ | NA | Retain |
| A21. Proportion of healthcare technicians | √ | √ | ○ | NA | Retain |
| A22. Proportion of professional technicians | √ | √ | ○ | NA | Retain |
| A23. Proportion of staffing | × | × | × | NA | Remove |
| A24. Proportion of senior positions and highly educated technicians | √ | ○ | × | Modify the name | Modify the name and remove at the country level |
| A3. Infrastructure, materials, and equipment | ‒ | ‒ | ‒ | NA | Retain |
| A31. Compliance rate of inspection equipment | √ | √ | ○ | Modify indicator evaluation criteria | Retain and modify indicator evaluation criteria |
| A32. Instrumentation uptime rate | × | × | × | Modify indicator evaluation criteria | Remove |
| A33. Informatization construction evaluation index | √ | × | × | NA | Remove at the city and country levels |
| A4. Laboratory capacity and safety | ‒ | ‒ | ‒ | NA | Retain |
| A41. Implementation rate of laboratory testing programs | √ | √ | ○ | NA | Retain |
| A42. Laboratory safety management | √ | √ | √ | NA | Retain |
| A43. Laboratory quality control coverage | √ | √ | √ | Modify indicator evaluation criteria | Retain and modify indicator evaluation criteria |
| A44. Class III biosafety lab | √ | ‒ | ‒ | NA | Retain |
| A5. Party construction | ‒ | ‒ | ‒ | NA | Retain |
| A51. Implementation of the party construction work responsibility system | √ | √ | √ | NA | Retain |
| B. Communicable disease prevention and control | ‒ | ‒ | ‒ | NA | Retain |
| B1. Overview of infectious disease prevention and control | ‒ | ‒ | ‒ | Adjust the scope of application of subordinate indicators | Retain and adjust the scope of application of subordinate tertiary indicators |
| B11. Comprehensive evaluation rate of information quality in epidemic reporting | ○ | ○ | ○ | NA | Retain |
| B12. Statutory infectious disease reporting rate in medical institutions | ‒ | ○ | ○ | NA | Retain |
| B13. Infectious disease surveillance completion rate | ○ | ○ | √ | Modify the name | Modify the name |
| B14. Outbreak standardized disposal index | × | ○ | √ | NA | Remove at the provincial level |
| B15. Coverage rate of direct network reporting of infectious diseases | ‒ | √ | √ | NA | Retain |
| B16. Reported incidence of class A and B statutory infectious diseases | ‒ | ‒ | ○ | Remove | Remove |
| B17. Timely response rate of automatic warning signals for infectious diseases | √ | √ | √ | NA | Retain |
| B18. Priority infectious disease surveillance completion rate | ○ | √ | ‒ | Modify the name | Modify the name |
| B19. Experimental diagnosis rate of priority infectious diseases | √ | √ | ○ | Modify indicator evaluation criteria | Retain and modify indicator evaluation criteria |
| B110. Laboratory testing capacity for priority infectious diseases | √ | √ | √ | Modify indicator evaluation criteria | Retain and modify indicator evaluation criteria |
| B111. Incidence of AFP cases in children under 15 years old | ○ | ○ | ○ | Modify indicator evaluation criteria | Retain and modify indicator evaluation criteria |
| B112. Severe respiratory syndrome monitoring | × | ○ | ○ | Modify indicator evaluation criteria | Modify indicator evaluation criteria and remove at the provincial level |
| B2. AIDS and syphilis prevention and control | ‒ | ‒ | ‒ | Add tertiary indicators | Retain and add subordinate tertiary indicators |
| B21. Coverage of interventions for high‒risk groups of AIDS | ‒ | √ | √ | NA | Retain |
| B22. Proportion of HIV‒infected and AIDS patients followed up with interventions | ‒ | ○ | ○ | NA | Retain |
| B3. Prevention and control of tuberculosis and leprosy | ‒ | ‒ | ‒ | Modify the name | Modify the name |
| B31. Incidence of tuberculosis | √ | √ | √ | NA | Retain |
| B32. Management rate of tuberculosis patients | √ | √ | √ | NA | Retain |
| B33. Supervisory coverage of tuberculosis control | ‒ | ○ | √ | Suggested removal | Retain |
| B4. Surveillance of insect‒borne infectious diseases | ‒ | ‒ | ‒ | NA | Retain |
| B41. Completion rate of vector monitoring | √ | √ | ○ | NA | Retain |
| B5. Endemic disease | ‒ | ‒ | ‒ | NA | Retain |
| B51. Completion rate of endemic disease monitoring | ○ | ○ | ○ | Modify indicator evaluation criteria | Retain and modify indicator evaluation criteria |
| B6. Immunization planning and vaccine management | ‒ | ‒ | ‒ | NA | Retain |
| B61. Childhood vaccination rate | √ | √ | √ | Modify indicator evaluation criteria | Retain |
| B62. Standardized treatment rate of suspected abnormal reactions to vaccination | √ | √ | √ | NA | Retain |
| B63. Children’s vaccination certification rate | ‒ | √ | √ | NA | Retain |
| B64. Coverage of full electronic vaccine tracing | √ | √ | √ | NA | Retain |
| B65. Monitoring completion rate for vaccine‒preventable infectious diseases | √ | √ | √ | Remove or Modify indicator evaluation criteria | Retain and modify indicator evaluation criteria |
| B66. Coverage rate of standardized vaccination clinics | √ | ○ | √ | NA | Retain |
| B67. Completion rate of population antibody level monitoring | ‒ | × | ‒ | Adjust the scope of application | Remove |
| C. Chronic non‒communicable disease prevention and control | ‒ | ‒ | ‒ | NA | Retain |
| C1. Overview of chronic non‒communicable disease prevention and control | ‒ | ‒ | ‒ | NA | Retain |
| C11. Coverage rate of whole‒population cause‒of‒death monitoring | √ | ○ | ‒ | NA | Retain |
| C12. Standardized registration and reporting rate of causes of death | √ | √ | √ | NA | Retain |
| C13. Target achievement rate of the National Healthy Lifestyle | × | × | ○ | NA | Remove at the provincial and city levels |
| C2. Monitoring of risk factors for chronic non‒communicable diseases | ‒ | ‒ | ‒ | NA | Retain |
| C21. Coverage rate of monitoring chronic disease risk factors | ○ | ○ | ‒ | NA | Retain |
| D. Public health emergency response | ‒ | ‒ | ‒ | NA | Retain |
| D1. Emergency disposal | ‒ | ‒ | ‒ | NA | Retain |
| D11. Regulated disposal index | √ | ○ | √ | NA | Retain |
| D12. Timely incident reporting rate | ‒ | **‒** | √ | Adjust the scope of application and modify indicator evaluation criteria | Retain and add at the provincial and city levels |
| D13. Information direct network reporting rate | ‒ | ‒ | √ | Adjust the scope of application | Retain and add at the provincial and city levels |
| D14. Emergency stockpile completeness rate | √ | √ | √ | Modify indicator evaluation criteria | Retain and modify indicator evaluation criteria |
| E. Health hazard monitoring and control | ‒ | ‒ | ‒ | NA | Retain |
| E1. Monitoring and control of occupational disease hazards | ‒ | ‒ | ‒ | NA | Retain |
| E11. Completion rate of priority occupational disease monitoring | √ | √ | √ | NA | Retain |
| E12. Occupational health monitoring rate | √ | √ | ○ | NA | Retain |
| E2. Foodborne disease prevention and control | ‒ | ‒ | ‒ | NA | Retain |
| E21. Completion rate of food safety risk monitoring | √ | √ | √ | NA | Retain |
| E3. Environmental hazards control | ‒ | ‒ | ‒ | NA | Retain |
| E31. Drinking water monitoring rate | √ | ○ | ○ | NA | Retain |
| E32. Air quality monitoring rate | × | × | × | NA | Remove |
| E4. Disinfection quality monitoring | ‒ | ‒ | ‒ | NA | Remove |
| E41. Coverage rate of disinfection quality monitoring | × | ○ | × | NA | Remove |
| F. Health education and promotion | ‒ | ‒ | ‒ | NA | Retain |
| F1. Health education for target groups | ‒ | ‒ | ‒ | NA | Retain |
| F11. Behavioral intervention index for target groups | × | × | ○ | NA | Remove at the provincial and city levels |
| F12. Awareness rate of key hygiene and disease prevention knowledge among target groups | × | ○ | ○ | NA | Remove at the provincial level |
| F13. Awareness rate of blood pressure and blood glucose in the population | × | ○ | ○ | NA | Remove at the provincial level |
| G. Information management | ‒ | ‒ | ‒ | NA | Retain |
| G1. Information gathering | ‒ | ‒ | ‒ | NA | Remove |
| G11. Evaluation index for timeliness and completeness of data reporting | ○ | ○ | ○ | Suggested removal | Remove |
| G2. Information utilization and analysis | ‒ | ‒ | ‒ | NA | Retain |
| G21. Evaluation index for data analysis | ○ | ○ | ‒ | NA | Retain |
| H. Skill and technical guidance | ‒ | ‒ | ‒ | Add tertiary indicators | Retain and add subordinate tertiary indicators |
| H1. Skill training | ‒ | ‒ | ‒ | NA | Retain |
| H11. Job skill training rate | √ | √ | √ | NA | Retain |
| H2. Technical guidance | ‒ | ‒ | ‒ | NA | Retain |
| H21. Coverage rate of grassroots professional guidance | √ | √ | ○ | NA | Retain |
| I. Integrated service indicators | ‒ | ‒ | ‒ | NA | Retain |
| I1. Other work capacities | ‒ | ‒ | ‒ | NA | Retain |
| I11. Completion rate of directive work | √ | √ | √ | Modify indicator evaluation criteria | Retain and modify indicator evaluation criteria |
| I12. Comprehensive evaluation of abilities and qualifications | √ | √ | √ | Modify indicator evaluation criteria | Retain and modify indicator evaluation criteria |
| I13. Project budget completion rate | √ | √ | √ | NA | Retain |
| J. Outcome of disease prevention and control | ‒ | ‒ | ‒ | NA | Retain |
| J1. Outcome indicators for communicable disease prevention and control | ‒ | ‒ | ‒ | NA | Retain |
| J11. Disability rate in newly discovered leprosy patients | ‒ | ‒ | √ | Adjust the scope of application and modify indicator evaluation criteria | Retain, modify indicator evaluation criteria, and add at the provincial and city levels |
| J12. Achievement rate of parasitic disease prevention and control goals | √ | √ | ○ | NA | Retain |
| J13. Compliance rate for communicable diseases under planning control | √ | √ | √ | NA | Retain |
| J14. Total incidence of infectious diseases | × | × | × | NA | Remove |
| J15. Mortality rate for statutory infectious diseases | √ | ○ | √ | NA | Retain |
| J2. Outcome indicators for chronic non‒communicable disease prevention and control | ‒ | ‒ | ‒ | NA | Remove |
| J21. Annual growth rate of health literacy | ○ | × | ○ | NA | Remove |
| J22. Formation rate of basic hygiene and disease prevention behaviors of the population | × | × | × | NA | Remove |
| K. Institutional development and satisfaction evaluation | ‒ | ‒ | ‒ | NA | Retain |
| K1. Scientific research capacity | ‒ | ‒ | ‒ | NA | Retain |
| K11. Comprehensive evaluation of scientific research projects | √ | ‒ | ‒ | Adjust the scope of application and modify indicator evaluation criteria | Retain, modify indicator evaluation criteria, and add at the city level |
| K2. Satisfaction evaluation | ‒ | ‒ | ‒ | NA | Retain |
| K21. Employee satisfaction | √ | √ | √ | NA | Retain |
| K22. Public satisfaction | ○ | √ | √ | NA | Retain |

“√” indicates suggested retention, “○” indicates consideration for exclusion, and “×” indicates definite exclusion. “‒” indicates that the indicators were not scored. For primary and secondary indicators, they were adjusted based on the screening of subordinate tertiary indicators and expert comments. For tertiary indicators, they did not need to be scored at the level that was not applicable. NA indicates that the experts did not comment on the corresponding indicators. AFP, acute flaccid paralysis.
